# Unlocking HER2 as a target in KRAS-mutated rectal cancer: a case of significant response to zanidatamab-based regimen

**DOI:** 10.3389/fimmu.2026.1925363

**Published:** 2026-08-19

**Authors:** Kexun Zhou, Yu Yang

**Affiliations:** Division of Abdominal Cancer, Department of Medical Oncology, Cancer Center, West China Hospital, Sichuan University, Chengdu, China

**Keywords:** biomarker-guided treatment, colorectal cancer, human epidermal growth factor receptor 2, KRAS mutation, zanidatamab

## Abstract

**Background:**

Colorectal cancer (CRC) with human epidermal growth factor receptor 2 (HER2) overexpression and RAS mutations is often associated with poor response to conventional treatments, presenting substantial therapeutic challenges. This report describes a remarkable response in a patient with HER2-amplified, KRAS-mutated metastatic rectal cancer, who was treated with a zanidatamab-based combination regimen.

**Case presentation:**

A 54-year-old male was admitted to our hospital complaining of hematochezia and was diagnosed with rectal adenocarcinoma via biopsy. Baseline imaging revealed extensive, unresectable liver metastases. Next-generation sequencing identified a KRAS G12D mutation alongside HER2 amplification. Although the combination of chemotherapy and bevacizumab yielded promising efficacy, but tumor markers still remained high. When the anti-HER2 bispecific antibody zanidatamab was added, after three cycles of this triplet therapy, the liver metastases exhibited radiographic regression, and tumor markers showed substantial decline. The patient achieved sufficient downstaging and subsequently underwent successful hepatic resection. Pathological examination confirmed negative surgical margins.

**Conclusion:**

This case underscores the potential of HER2 inhibition as an effective therapeutic strategy in KRAS-mutated CRC. It also highlights zanidatamab’s promise as part of a conversion therapy approach in patients with historically refractory disease.

## Introduction

Colorectal cancer (CRC) ranks as the third most commonly diagnosed malignancy globally, with persistently high incidence and mortality rates (1). Despite significant advancements in early screening and multimodal therapies, the prognosis for patients with advanced CRC remains poor, particularly in subsets harboring specific molecular alterations (2, 3).

CRC is characterized by profound molecular heterogeneity, with HER2 amplification/overexpression (observed in 3–5% of cases) correlating with aggressive clinicopathological features (2). Robust evidence indicates that HER2-positive CRC is associated with deeper tumor invasion, higher rates of lymph node metastases, and earlier distant dissemination, collectively contributing to inferior clinical outcomes (4). Moreover, these patients exhibit diminished responses to standard chemotherapy regimens and intrinsic resistance to anti-EGFR antibodies, likely due to cross-activation between HER2 and EGFR signaling pathways (5).

While anti-HER2 targeted therapies have demonstrated modest efficacy in later-line treatment of HER2-positive CRC, their utility in RAS-mutant cohorts remains strikingly limited. The MyPathway trial evaluated dual HER2 blockade in CRC, reporting an overall response rate (ORR) of 32%, with median progression-free survival (PFS) and overall survival (OS) of 2.9 and 11.5 months, respectively. Notably, subgroup analyses revealed marked disparities. Only 1 of 13 KRAS-mutant patients responded (ORR, 7.7%), compared to 17 of 43 KRAS-wildtype patients (ORR, 40%). These findings underscore the critical unmet need for novel strategies in this refractory population (6).

Zanidatamab is a novel humanized bispecific HER2-targeted monoclonal antibody that promotes HER2 clustering and internalization, suppresses HER2 signaling, and activates immune-mediated anti-tumour responses, leading to potent activity across diverse HER2 expression levels. The HERIZON-BTC-01 study led to its approval for HER2-high (IHC 3+) unresectable or metastatic biliary tract cancer (7). Promising activity has also been demonstrated in HER2-positive advanced gastroesophageal cancer, with a phase III trial (NCT05152147) demonstrating an ORR of 70.7% and robust survival improvement when zanidatamab combined with immunotherapy and chemotherapy as first-line treatment (8). However, no clinical data currently exist regarding the efficacy of zanidatamab in HER2-amplified, RAS-mutant CRC.

Herein, we present a case of metastatic rectal adenocarcinoma with concurrent KRAS G12D mutation and HER2 amplification that achieved significant liver metastasis regression following zanidatamab combined with FOLFOX and bevacizumab. This report contextualizes therapeutic advances for HER2-driven CRC while highlighting a potential paradigm for overcoming RAS-mediated resistance.

## Case presentation

**Figure 1:**
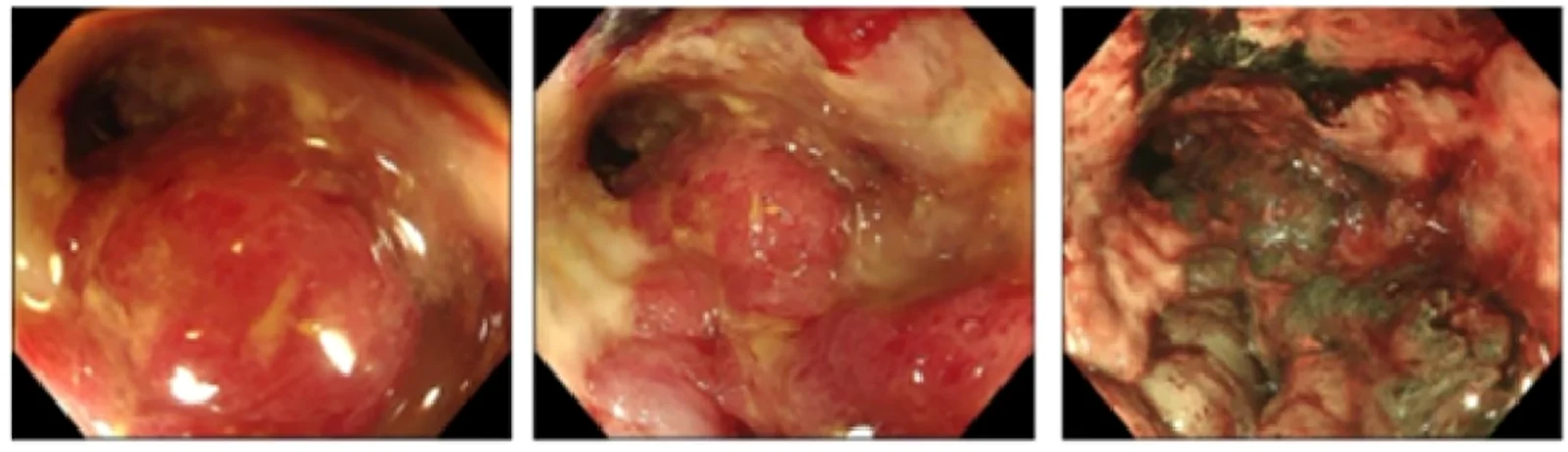
Colonoscopy of rectal. At 5 cm proximal to the anal verge, a circumferential ulcerated mass with heaped-up margins is noted, causing complete luminal obstruction. The tumor exhibits characteristic friability upon contact, suggesting high-grade malignancy.

In June 2025, a 54-year-old male visited our hospital complaining of intermittent hematochezia and tenesmus. Colonoscopy revealed a circumferential ulcerative mass located 5 cm from the anal verge, causing luminal stenosis that precluded endoscopic advancement (**Figure 1**). Histopathological examination of biopsy specimens demonstrated adenocarcinoma with high-grade intraepithelial neoplasia. Immunohistochemistry (IHC) showed MLH1 (+), MSH2 (+), MSH6 (+), PMS2 (+) and HER2 (3+). Next-generation sequencing (NGS) identified a KRAS p.G12D mutation (variant allele frequency: 1.16%) and ERBB2 amplification (copy number: 28.7).

**Figure 2:**
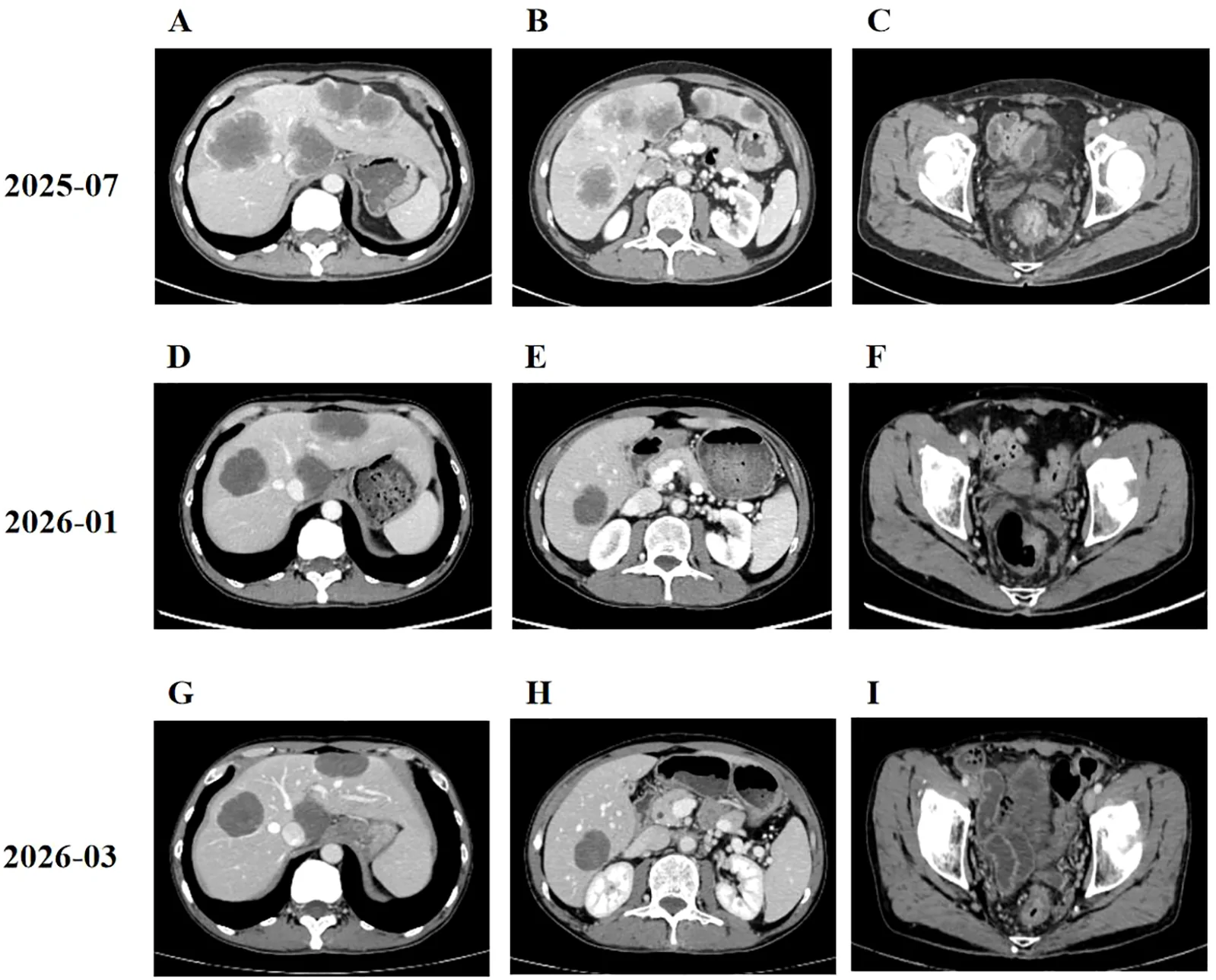
Imaging examination of liver mass and primary lesion. Abdominal CT showing the radiological response to therapy. **(A–C)** advanced rectal cancer with liver and lymph nodes metastases at the initial diagnosis. **(D–F)** partial response was achieved after six cycles of CAPOX+Bevacizumab treatment. **(G–I)** continued partial response after four cycles of FOLFOX+Bevacizumab+Zanidatamab treatment.

**Figure 3:**
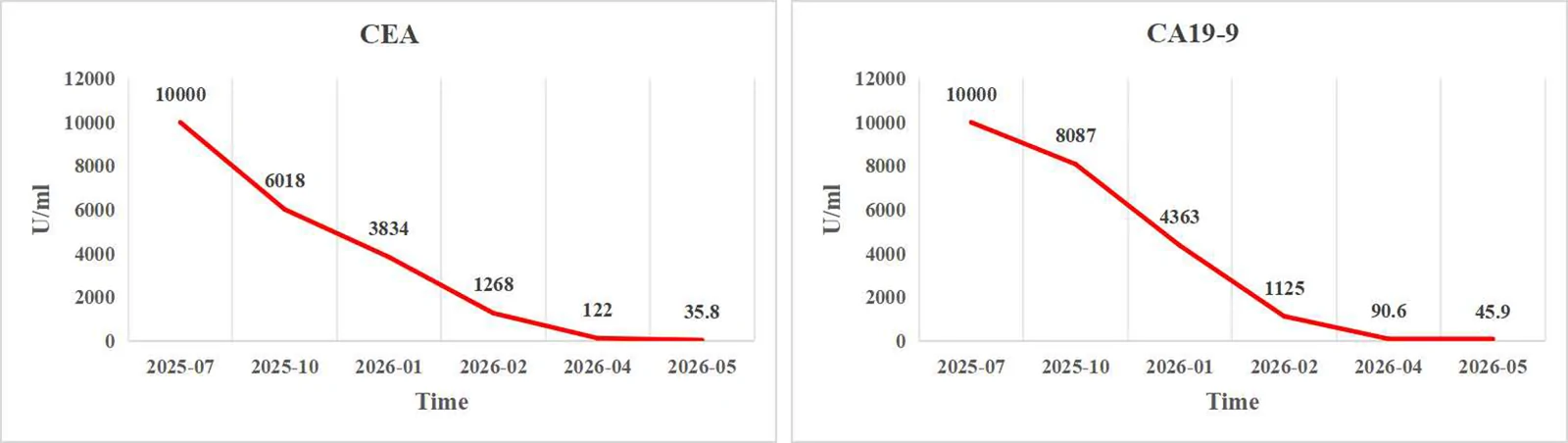
The trend of serum CEA and CA19-9.

Enhanced computer tomography (CT) of the abdomen demonstrated advanced rectal cancer with mesorectal, left iliac nodal involvement, and multiple liver metastases (**Figure 2**). As complete resection could not be obtained due to the extensive metastasis, systemic therapy was recommended by a multidisciplinary team (MDT). First-line therapy with CAPOX plus bevacizumab (oxaliplatin 130 mg/m² on day 1+bevacizumab 7.5 mg/kg on day 1+capecitabine 1000 mg/m² bid on days 1-14, q3w) was administrated. After six cycles of treatment, local imaging assessment indicated a partial response (PR) (according to RECIST version 1.1) (**Figure 2**), but tumor markers still remained high (**Figure 3**).

Reassessment by the MDT concluded unresectability due to persistent multifocal liver metastases. The MDT recommended intensifying the chemotherapy backbone to FOLFOXIRI (fluorouracil, leucovorin, oxaliplatin, and irinotecan) as a conversion strategy, while the addition of concurrent targeted therapy was to be decided later, contingent on the patient’s tolerance. Ultimately, the patient declined FOLFOXIRI due to concerns about toxicity. After further discussion, the patient opted for a combination of FOLFOX (oxaliplatin 85 mg/m² on day 1, leucovorin 400 mg/m² on day 1, fluorouracil 400 mg/m² bolus followed by 2400 mg/m² continuous infusion over 46 hours) with bevacizumab (5 mg/kg on day 1) and zanidatamab (20 mg/kg on day 1) every 2 weeks. Treatment-related grade 3 diarrhea (according to CTCAE version 5.0), requiring loperamide intervention, resolved without therapy discontinuation. By March 2026, serial imaging revealed continued PR with increased necrotic components (**Figure 2**), accompanied by a substantial decrease in carcinoembryonic antigen levels (**Figure 3**).

**Figure 4:**
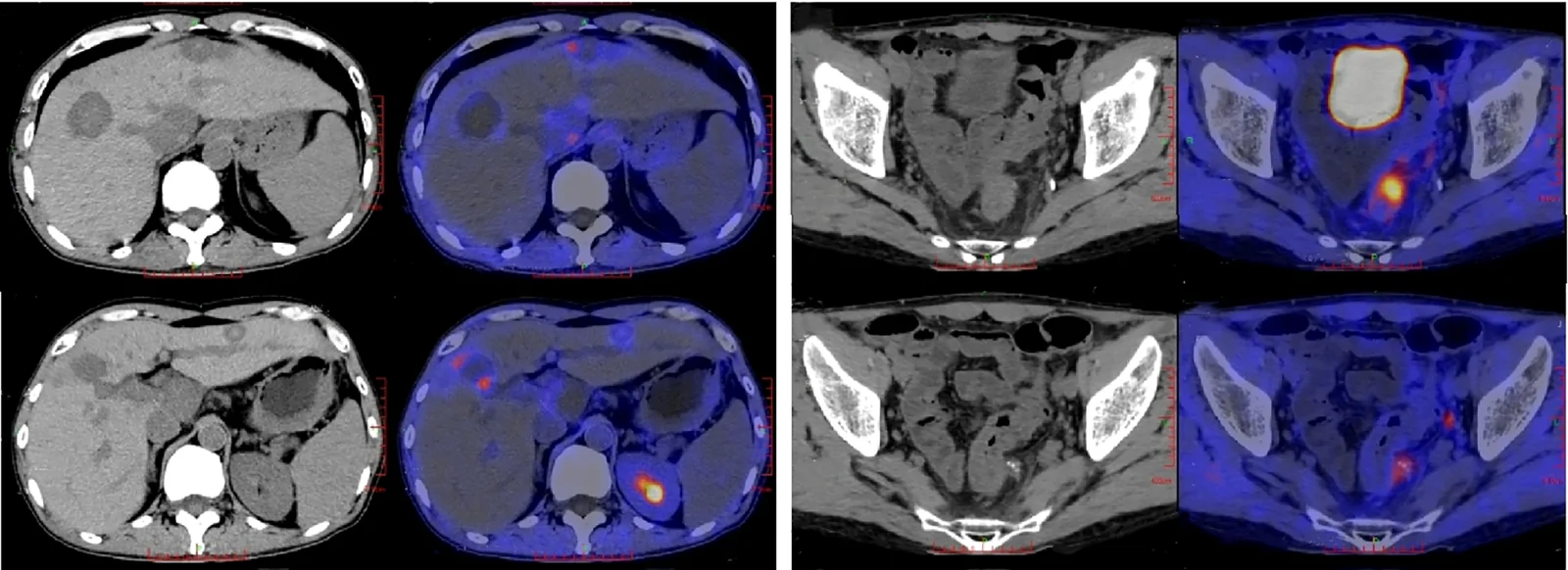
FAPI PET-CT.

**Figure 5:**
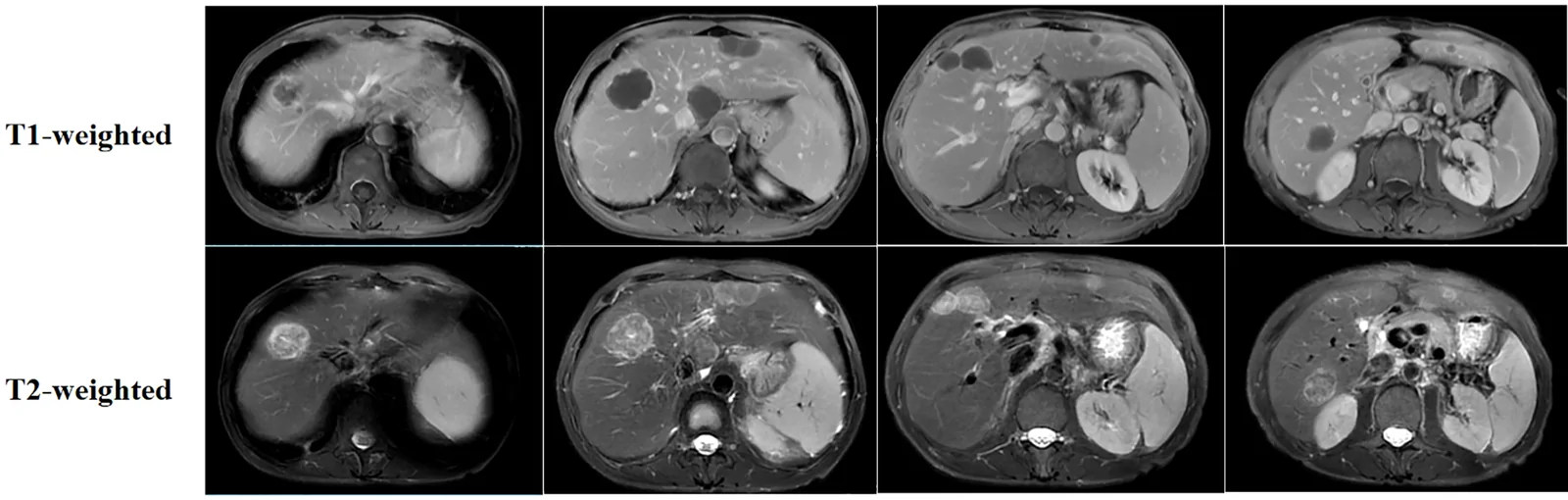
Liver magnetic resonance imaging before surgery.

Following six cycles of FOLFOX combined with bevacizumab and zanidatamab (last administration on April 28, 2026), FAPI PET-CT was performed on May 18, 2026 (**Figure 4**). The imaging findings indicated a favorable response and prompted reassessment of surgical candidacy. The MDT discussion was subsequently convened, and liver magnetic resonance imaging (MRI) was recommended to provide complementary anatomical information regarding the hepatic lesions, including precise number, location, and spatial relationship with critical vascular structures. Accordingly, liver MRI was performed on June 2, 2026 (**Figure 5**). The detailed anatomical mapping obtained from MRI was then integrated into the surgical decision-making process, facilitating individualized operative planning. Based on these findings, the patient underwent hepatic tumor resection on June 23, 2026. Postoperative pathological diagnosis revealed adenocarcinoma with treatment response (tumor regression grade 2). Combined with the clinical history and morphological features, the findings were consistent with rectal cancer metastasis. Surgical margins were negative.

## Discussion

This study reports the first case of a metastatic rectal cancer patient with concurrent HER2 amplification and KRAS G12D mutation, who achieved significant tumor shrinkage and successful conversion to a resectable state after treatment with zanidatamab combined with FOLFOX chemotherapy and bevacizumab. This finding offers a groundbreaking therapeutic approach for KRAS-mutant mCRC patients, traditionally considered resistant to HER2-targeted therapies, and provides important insights for the broader field of solid tumor treatment.

Traditional views suggest that KRAS mutations lead to acquired resistance to HER2-targeted therapies by continuously activating downstream signaling pathways, a phenomenon confirmed across various cancer types. In pancreatic ductal adenocarcinoma, Barzi et al. demonstrated that acquired KRAS mutations, accompanied by loss of ERBB2 amplification, are key mechanisms of resistance to HER2-targeted treatment (9). Similarly, in breast cancer, KRAS mutations are a major cause of primary resistance to trastuzumab (10), and studies in upper gastrointestinal cancers also suggest poor outcomes for HER2-positive patients with KRAS mutations treated with trastuzumab (11, 12).

HER2 amplification occurs in approximately 3-5% of colorectal cancers, mostly in RAS wild-type tumors (2). Reviewing the development of HER2-targeted therapy in mCRC, Bertotti et al. found that HER2-positive patients were sensitive to combination therapy with trastuzumab and lapatinib but not to single-agent treatment. The HERACLES trial, a multicenter phase II study, evaluated the efficacy of trastuzumab and lapatinib in KRAS wild-type and HER2-overexpressing mCRC patients who were resistant to standard therapies. The study showed an objective response rate (ORR) of 30%, a mPFS of 5.0 months, and a mOS of 11.5 months, respectively (13). In the HERACLES-B study, patients treated with a combination of pertuzumab and trastuzumab showed an ORR of 9.7%, with a low toxicity profile and similar mPFS to other HER2-targeted regimens (14). The MyPathway trial revealed a significant difference in efficacy between KRAS-mutant and wild-type groups, reinforcing the recognition of KRAS mutations as resistance markers (6). As a result, current treatment guidelines recommend HER2-targeted therapy primarily for RAS wild-type mCRC patients (15).

The observed response to anti-HER2 therapy in this KRAS G12D-mutated patient may be attributed to the low mutant allele frequency. Variant allele frequency (VAF) reflects the proportion of tumor cells harboring a specific mutation, and a low VAF suggests that the KRAS G12D mutation likely exists only in a small subclonal population, while the majority of tumor cells retain wild-type KRAS status. This molecular characteristic aligns with multiple clinical observations. Previous study indicated that in EGFR-targeted therapy, patients with KRAS mutation VAF below 30% achieved a disease control rate of 44.4%, significantly higher than those with higher VAF (P = 0.038) (16). Similarly, a phase II trial demonstrated that RAS mutations with VAF <5% did not significantly impair response to EGFR inhibitors (17). Furthermore, the basket trial of trastuzumab deruxtecan (T-DXd) showed therapeutic responses in KRAS-mutant CRC patients (18), collectively suggesting that low VAF KRAS mutations may not completely abrogate the efficacy of targeted therapies. Potential underlying mechanisms include predominant expression of wild-type KRAS, interclonal regulatory interactions, or insufficient downstream pathway activation by mutant KRAS. These findings not only provide a plausible explanation for this case but also underscore the importance of incorporating mutational burden assessments into therapeutic decision-making for precision oncology.

Zanidatamab is the first approved HER2-targeted bispecific antibody. Unlike traditional therapies like trastuzumab or pertuzumab, zanidatamab targets two non-overlapping epitopes of the HER2 protein, forming a unique ‘cap’ conformation that not only more effectively blocks signaling pathways but also enhances complement-dependent cytotoxicity (CDC) and antibody-dependent cell-mediated cytotoxicity (ADCC) (19, 20). This mechanism could explain the efficacy of zanidatamab in KRAS-mutant mCRC patients. Clinical studies strongly support its superior efficacy. A phase I trial of zanidatamab in HER2-positive mCRC patients, previously treated with standard therapies, showed a PFS of 6.8 months, with an ORR of 38%, even in 25% of patients who had failed prior HER2-targeted therapies. These results outperformed the trastuzumab/pertuzumab (dual-HER2) combination (21). Additionally, a phase II study of zanidatamab combined with FOLFOX ± bevacizumab in HER2-positive mCRC patients demonstrated an ORR of 100%, with all eleven evaluable patients achieving partial response (22). In terms of safety, zanidatamab exhibited favorable tolerability, with only 1% of patients experiencing grade 3 or higher adverse events. Although the case presented here involved a grade 3 diarrhea, it was manageable with symptomatic treatment, and the patient tolerated the therapy well.

Despite these encouraging results, the inherent limitations of this single-case analysis must be acknowledged. Going forward, several critical research priorities emerge. First, the development of refined predictive biomarker algorithms is essential to accurately identify responsive patient subsets. Second, systematic investigation into optimal combination regimens warrants dedicated study. Finally, delineating the precise molecular mechanisms by which bispecific antibodies circumvent KRAS-mediated resistance is of paramount importance. Encouragingly, DESTINY-PanTumor02 trial indicated that next-generation antibody-drug conjugates (ADCs), such as deruxtecan, show promise for some KRAS-mutant patients, offering a potential redefinition of the patient population suitable for HER2-targeted therapy (23).

In conclusion, this case provides preliminary clinical evidence supporting the potential therapeutic value of HER2-targeted therapy in HER2-amplified, KRAS-mutated metastatic CRC. Although limited to a single case, these findings generate a clinically relevant hypothesis and support further investigation of zanidatamab-based regimens in this challenging molecular subtype.

## Data Availability

The raw data supporting the conclusions of this article will be made available by the authors, without undue reservation.
